# Neuroprotective and Therapeutic Strategies against Parkinson’s Disease: Recent Perspectives

**DOI:** 10.3390/ijms17060904

**Published:** 2016-06-08

**Authors:** Sumit Sarkar, James Raymick, Syed Imam

**Affiliations:** Division of Neurotoxicology, National Center for Toxicological Research/FDA, Bldg. 53D, HFT-32, Jefferson, AR 72079, USA; James.Raymick@fda.hhs.gov (J.R.); syed.imam@fda.hhs.gov (S.I.)

**Keywords:** Parkinson’s disease, dopamine, l-DOPA, striatum, substantia nigra, deep brain stimulation, olfaction

## Abstract

Parkinsonism is a progressive motor disease that affects 1.5 million Americans and is the second most common neurodegenerative disease after Alzheimer’s. Typical neuropathological features of Parkinson’s disease (PD) include degeneration of dopaminergic neurons located in the pars compacta of the substantia nigra that project to the striatum (nigro-striatal pathway) and depositions of cytoplasmic fibrillary inclusions (Lewy bodies) which contain ubiquitin and α-synuclein. The cardinal motor signs of PD are tremors, rigidity, slow movement (bradykinesia), poor balance, and difficulty in walking (Parkinsonian gait). In addition to motor symptoms, non-motor symptoms that include autonomic and psychiatric as well as cognitive impairments are pressing issues that need to be addressed. Several different mechanisms play an important role in generation of Lewy bodies; endoplasmic reticulum (ER) stress induced unfolded proteins, neuroinflammation and eventual loss of dopaminergic neurons in the substantia nigra of mid brain in PD. Moreover, these diverse processes that result in PD make modeling of the disease and evaluation of therapeutics against this devastating disease difficult. Here, we will discuss diverse mechanisms that are involved in PD, neuroprotective and therapeutic strategies currently in clinical trial or in preclinical stages, and impart views about strategies that are promising to mitigate PD pathology.

## 1. Introduction

It is only in last few decades that some breakthrough in development of specific therapies has been made to treat PD patients since the first patient was diagnosed with Parkinsonian syndrome [1]. The aim of this review is to enumerate and highlight developments that occurred in last few decades and also review the advantages and disadvantages of those anti-parkinsonian therapies in the years to come.

It was in 1960s when it was discovered that dopamine present in the striatum is responsible for motor symptoms of PD [2,3]. Until then, most PD treatments revolved around replacing dopamine, thus ameliorating main pathological features such as slow movement, rigidity and resting tremor. Levodopa was introduced after it was demonstrated that main cause of PD is due to degeneration of dopaminergic neurons of the substantia nigra [4,5] and for the last several decades Levodopa prescription has been the standard therapy for PD patients although several complications have arisen since Levodopa can generate other motor complications as well [6,7,8].

Due to the complications associated with levodopa, new drugs in the form of dopamine receptor agonists have been developed for patients who have the early stages of PD; these dopamine receptor agonists confer anti-PD therapy with relatively low risk of developing dyskinesia [9,10,11]. The efficacy of these drugs in treating PD was demonstrated in subsequent large-scale clinical trials using ropinirole and pramipexole [12,13]. Later, it was found that the dopamine receptor agonists cause more severe non-motor side effects than levodopa such as mood swing, sleep attacks, and fatigue [14]. Therefore, it is concluded that the motor and non-motor side effects in various therapies that use dopamine receptor agonists showed only partial recovery during disease progression and is no longer ideal for anti-parkinsonian therapy.

Recent major breakthroughs to treat PD patients came in the form of surgical treatment. These treatment modalities were based on intervention of basal ganglia circuitry [15,16,17]. Initially, surgeries were targeted to internal globus pallidus (GPi) or the subthalamic nucleus (STN) but most recently deep brain stimulation procedures have been adopted to treat PD patients [18,19]. Although surgical treatment alleviates motor symptoms seen in PD patients, recent studies revealed the side effects of this procedure may lead to non-motor complications that may have occurred due to misplaced DBS electrodes.

Although dopaminergic cell/tissue grafting has recently been under discussion to treat PD patients [20], recent large clinical trials [21,22] have suggested it is minimally effective and can cause unexpected side effects, thus putting this method of treatment in serious doubt. Treating PD patients using stem cells is another lucrative approach; however, due to safety and regulatory issues [23], this approach may not be feasible and will be discussed later in this review.

Currently, most of the therapeutics for PD treatment and therapeutics under development target dopaminergic motor symptoms. However, we cannot rule out the possibility that not only PD pathology is confined to the degeneration of dopaminergic neurons but also include non-motor such as cognitive impairment, hallucinations and other disturbances due to dysfunction of autonomic nervous system also play major role(s) in the array of impairments seen in PD patients [24,25,26,27,28,29,30]. Therefore, during development of pharmacotherapeutics, the focus should be to treat non-motor symptoms while also reducing parkinsonian motor signs. To date, although several anti parkinsonian therapeutics showed promise in providing neuroprotective therapy against this devastating debilitating disorder in preclinical animal studies [31,32,33], none of them has been successful in humans. Progress in neuroprotective therapy has been hindered partly due to the lack of an adequate model and partly due to the lack of reliable markers that would allow us to detect early disease symptoms. Moreover, the endpoints need to be chosen carefully so that they are confounded by the symptomatic effects of PD therapeutics [34,35].

In this review, the term “neuroprotection” means therapies that can confer neuroprotection to cell especially dopaminergic neurons of the midbrain. Although numerous theories have been proposed and several neuroprotective agents have been used in the clinical trials, none of them were established as a neuroprotective agent. Thus, further research is necessary to develop such an agent that will qualify as a “neuroprotective” agent that will mitigate PD pathology.

## 2. Neuropathology of PD

Parkinsonism is a progressive motor disease that affects 1.5 million Americans. It is the second most common neurodegenerative disease after Alzheimer’s and PD affects close to 5% of the population that are over 65 years old. The degeneration of dopaminergic neurons in the substantia nigra lead to PD [36]. Due to abnormal protein folding and ER stress, a toxic protein named Lewy bodies are formed that are commonly observed in PD patients. This toxic protein, Lewy bodies, is made of different proteins such as α-synuclein [37,38], synphilin-1 [39], and ubiquitin [40]. Lewy bodies have also been observed in other areas of the brain such as hind brain, spinal cord and enteric nervous system. Lewy bodies first appear in the periphery [41], subsequently it travels to brain stem and eventually in the cortex. Although there is some controversy as to whether all PD patients do have α-synucleinopathy, all α-synucleinopathies have one thing common and that is pathology found in discrete location and clinical manifestations may be due prionopathy that arose from different regions of the brain [22]. Thus, a misfolded α-synuclein can trigger a downstream cascade of events that may lead to clinical symptoms. There are other forms of PD that involve abnormally phosphorylated tau such as progressive supra nuclear palsy, cortico-basal degeneration, parkinsonism-dementia complex, and fronto-temporal dementia with Parkinsonism. Recent evidence reveals that systemic dysfunction of neurons can lead to continuous degeneration of cells and possibly produces pathophysiological stress. Finally, it is possible that age and environmental factors together induce cellular stress leading to failure of compensatory mechanism(s) and ultimately clinical Parkinsonism with chronic neuronal dysfunction [42].

## 3. Striatal Pathophysiology of PD

It is customary to focus on the striatonigral pathway since the majority of the dopaminergic neurons in the SNpc are lost in PD. The neurons in the SNpc produce dopamine that goes to the caudate putamen and main nucleus of the basal ganglia. The basal ganglia consist of nuclei that are involved in motor and cognitive action selection. Dopamine from the striato-nigral pathway regulates both cortex and thalamus that are synaptically connected with striatal interneurons and spiny projection neurons. Around 90% of the striatal cells are spiny projected neurons and inhibitory and they have either D1 or D2 dopamine receptors and innervate distinct target nuclei [43]. A recent optogenetic study confirms that the neurotransmitter dopamine mainly regulates motor activity by stimulating the neurons in the nigra that project to the striatum and mostly express D1 spiny neurons rather than neurons of the pallidum that project to the striatum, which mostly controls the excitation of the thalamo-cortical pathway [44]. Thus, reduction of the main neurotransmitter dopamine in the striatonigral pathway presumably imbalances the striatal output so that it impairs thalamic excitation of the cortex which eventually affect the normal movement of the arms. Correcting motor deficits by stimulating both pathways may be theoretically sound; however, this approach can increase dopamine amount and motor dysfunction can worsen when a D2 receptor antagonist is used in conjunction with levodopa. Conversely, a different response has been observed when levodopa was used alone as oppose to D2 receptor agonist treatment that have been used to prolong the start of levodopa therapy [45,46]. It is important to note that spiny neurons present in the corpus striatum must relay information from discrete regions of the brain, along with their receptors including cortical, thalamic and nigral neurons. All of these pathways have been reported to be destroyed in an experimental PD model [43]. Therefore, it is plausible that cognitive and motor impairments commonly seen in PD patients are due to destruction of relay of information via striatonigral synaptic pathway rather than just only a decrease in dopamine. Another plausible mechanism is that aberrant striatal function could lead to axon degeneration and eventually result in neuronal loss [47,48]. The recent studies from experimental PD models and human clinical trials support the notion that PD pathology originates in the axon terminal [49].

## 4. Mechanisms of PD Pathogenesis and Possible Targets for Neuroprotection

To confer neuroprotection against Parkinson’s disease, it is imperative to understand the main mechanisms that are involved in development and progression of the disease pathology. In PD, several genetic and environmental factors and their interplay are involved in generating PD pathogenesis. The mechanisms that could contribute to the PD pathogenesis are: reactive oxygen species production through cellular stress, dysfunction of mitochondria, abnormal protein folding due to ER stress, abnormal cytoplasmic protein inclusions, neuroinflammation, cell death and loss of trophic factors (GDNF, neurturin, *etc.*) (Table 1). It is likely that no one mechanism is responsible for the generation of PD; instead, several molecular pathways acting together in a network to induce degeneration of dopaminergic neurons. We will discuss briefly each mechanism that is pertinent for the development neuroprotective therapies.

### 4.1. Oxidative Stress and Mitochondrial Dysfunction

Reactive oxygen species (ROS), if produced in low concentration, are helpful in regulating cell function as they trigger subsequent important signaling event but when it is produced in excess it can overwhelm antioxidant defenses and may cause oxidative stress [51]. Thus, these toxic free oxy radicals can attach with other cellular components and destroy normal cellular functions. The brain requires constant oxygen at a significantly high rate; thus, the presence of these free radicals and low amounts of antioxidant molecules present in the brain make the brain more vulnerable to oxidative damage [52]. Increased amount of lipid peroxidation and molecules related to oxidative stress has been demonstrated in the human PD brain [53,54].

Complex I inhibition has been documented in the substantia nigra of humans afflicted with PD [55], and other complex I inhibitors including MPP+ and rotenone have been documented in *in vivo* model of PD [56,57,58]. Although the exact mechanism of mitochondrial dysfunction is not known, it is plausible that mutation of either inherited or acquired mutations in mitochondrial DNA are responsible [59,60,61]. It is also plausible that excessive iron content as seen in human PD could induce cell death due to presence of free oxidative radicals [54,62].

Glutathione, an antioxidant protein found in its reduced form in the mid brain substantia nigra of human PD [63,64,65] suggesting damage of the anti-oxidative system in human. Recently, it has been demonstrated in humans that PTEN-induced putative kinase (PINK1) and DJ-1 [66,67,68] are the genes involved in inducing familial forms of PD and also involved in reducing stress due to oxidative free radical production. Several neuroprotective agents that target different pathways have been proposed. These putative neuroprotective agents are either MAO inhibitors, or agents that can increase the electron transport, *i.e.*, CoQ and chemicals such as selenium that can confer protection against free radical in the cell. The major reason that they are useful is that they have few adverse effects, although clinical trials to evaluate their efficacy are still lacking [69].

### 4.2. Protein Aggregation and Misfolding

In mammalian cells, endoplasmic reticulum (ER), a cytosolic compartment plays an important role in protein folding. Once peptides are synthesized in the cytoplasm, it gets transported to ER where chaperones in the ER keep them in proper shape. However, due to mutation, overexpression or unusual post-translational modifications misfolding ensues. When this happens, stress in the ER occurs. When the ER is severe, the cell death program gets activated. Like any other cell, neurons are also susceptible to effects of misfolded proteins or other mutant proteins available in the cell. As we know, neurons are post-mitotic cells that usually depend on protein quality control and stress responses such as unfolded protein response (UPR) so that they can adapt and restore homeostasis. In neurodegenerative disorders like PD, the unusual aggregation and accumulation of misfolded proteins are found in the ER and these proteins are harmful to neurons. In human PD, role of UPR has been investigated and it has been reported mainly in the autosomal recessive form of PD due to mutation of the parkin gene. The parkin gene mainly controls ubiquitin ligase function of the protein [70,71]. Thus, when ubiquitin ligase activity lost, it leads to aggregation of parkin and subsequent ER stress and cell death [72]. Recently, two markers of ER stress pPERK and pEIF2α have been observed in neurons of substantia nigra of human that afflicted with PD [70]. In human PD, pPERK immunopositive neurons were co-localized with α-synuclein. These findings suggest a positive correlation between ER stress in the dopaminergic neurons and misfolded protein α-synuclein. The ability of trehalose to prevent protein aggregation and tyrosine hydroxylase (TH) loss *in vivo* was tested in an MPTP induced model of PD [73].

Recent evidence suggests there are two proteins that are linked to genetic forms of PD such parkin and ubiquitin carboxyl-terminal hydrolase L1 (UCH-L1), and further confirms the association between formation of misfolded protein and development of PD [71,74]. Recently, it has been demonstrated that key substrates play an important role in protein turnover and degradation and it is HSP70 that has the capability to modulate toxicity induced by α-synuclein [75,76]. On the other hand, UCH-L1 acts as an enzyme that recycles ubiquitin in neurons and destruction of UCH-L1 can lead to formation of misfolded proteins [77,78,79].

Thus, all of the aforementioned evidences suggest that overwhelming amount of α-synuclein or lack of clearance of α-synuclein and further aggregation of other misfolded proteins could be the key mechanisms that lead to PD. Thus, neuroprotective therapies should be aimed at the prevention of aggregation of misfolded proteins; simultaneously, we should also investigate agents such as enhancers of parkin or UCH-L1 that have the ability to clear such misfolded proteins from the cell as they may also confer neuroprotection.

### 4.3. Neuroinflammation

One of the key features of PD pathology is neuroinflammation [80,81,82]. A plethora of studies has demonstrated the activation of microglia in the SN and striatum of PD inflicted humans and PD animal models [73]. It has also been found that pro-inflammatory cytokines such as IL-1beta, IL-6, and TNF-α are increased in the CSF and basal ganglia of PD patients [83,84,85]. Additionally, recent evidence has suggested that a complementary system may also play a role in PD pathogenesis as elevated levels of complement proteins in Lewy bodies were observed in PD [86,87]. Although the underlying mechanism of microglial activation in PD not well elucidated, it is likely that the pro-inflammatory cytokines and toxic α-synuclein can activate microglia in the brain [81]. In *in vitro* studies, it has been recently been reported that different chemical conformation such as nitrated and aggregated forms of α-synuclein can induce microglial activation and eventually release more chemicals that are toxic to cells [88,89,90,91]. In a mouse model of PD, it has been shown that α-synuclein or modified forms of protein can induce both microglial and humoral responses [92,93], and when NF-KB signaling was inhibited by a chemical, it can protect the dopaminergic neurons [94].

Currently, several putative neuroprotective agents that have shown in either an animal model or in an *in vitro* model are considered as anti-neuroinflammtory. Non-steroidal anti-inflammatory agents are commonly used to treat pain and to treat inflammation; however, in animal studies as well in *in vitro*, it has been shown that they can also prevent the degeneration of dopaminergic neurons [80]. It is important to note that recent epidemiological studies have demonstrated that certain NSAIDs and statins can significantly mitigate the risk of having PD [95,96,97,98]. One such agent is minocycline, which has anti-inflammatory potential and is currently being investigated in humans [99].

### 4.4. Excitotoxicity

A mechanism that has not been well documented in the pathogenic mechanism of PD is excitotoxicity. Glutamate is the key neurotransmitter in mammalian CNS and a main player in the excitotoxic process. It is well established that glutamate receptors are present in abundance in the dopaminergic neurons of the SN and they are innervated by glutamate that came from thalamus and cortex. To induce cell death, glutamate may increase the intra-cellular calcium level following NMDA receptor activation [100] followed by peroxynitrite production [101]. Thus, it is quite normal that 3-Nitrotyrosine, has been found to be abnormally higher in SN of PD patients [102]. When NMDA receptor antagonists were used in an MPTP induced model, it was found to be neuroprotective against dopaminergic cell loss in SN [103,104]; however, the antagonists have limited use due to its low potency and low tolerability. Riluzole has glutamate antagonistic properties but did not confer neuroprotection in a small clinical trial conducted in humans [105]. Along the same line, amantadine showed moderate glutamate antagonistic properties and other actions as well [106]. The most promising glutamate antagonist to protect dopamine neurons against PD should be selective for specific channel subunits to confer neuroprotection [107].

### 4.5. Apoptosis

Apoptosis plays a key role in development and in neural injury. Although the role of apoptosis in PD has been controversial, a plethora of research studies involving human PD brains demonstrated both apoptotic and autophagic cell death [108,109,110]. Cell death due to apoptosis has been well established in *in vivo* PD models [111,112]. Although there are several theories have been proposed that may activate the apoptotic pathways, oxidative stress, protein aggregation and excitotoxicity are the key processed that are involved in cell death. Thus, conceivably, agents that can inhibit the apoptotic or cell death pathways could be used as neuroprotective agents in PD. Recently, two such compounds that have the ability to prevent these pathways are currently being investigated tested in clinical trials [22,113].

### 4.6. Loss of Trophic Factors

Recently, it has been established that neurotrophic factors play an important role in the survival of cells, and lack of these factors can trigger cell death pathways in PD as well. These factors such as BDNF [114], GDNF [115], and NGF [114], have all been found to be dramatically decreased in the nigra of human brain inflicted with PD. Therefore, treatment with growth factors could be another option that we can use to mitigate PD pathology. Since the growth factors are able to stimulate growth and arborization of dopaminergic neurons, they could be ideal as neuroprotective agents. In animal models of PD, it has been shown that both GDNF and neurturin are able to restore dopaminergic loss against chemically induced PD [116,117,118]. While the clinical trial using GDNF has been concluded in human trials [119,120], neurturin is still in Phase II trial.

## 5. Neuroprotection in Clinical Trials: Recent Updates

Although a plethora of putative drugs have shown promise in animal models of PD, the results have not translated into therapies that can be used for neuroprotection in human PD. The main hurdles to establish a neuroprotective strategy are: the inherent complexity of the clinical tools that are currently available to evaluate the disease progression, the precise time when one should start this kind of drug as an intervention and last but not the least whether this kind of intervention has any beneficial effect.

### 5.1. Levo-Dopa as a Neuroprotective Agent

The various current treatment strategies for PD mostly focus on (a) non-pharmacological methods such as exercise, dancing, *etc.*; (b) pharmacological approaches that include both dopamine replacement therapy and non-dopaminergic approaches; and (c) surgical approaches such as deep brain stimulation, stem cell replacement, *etc.*

The loss of dopaminergic neurons and resulting deficit in PD was a significant lead for the development of dopamine replacement based therapies to restore function. Such attempts led to the discovery of levodopa (l-DOPA, l-3,4-dihydroxyphenylalanine) and various other dopamine receptor agonists. These dopamine replacement therapies provide symptomatic relief during the disease and help alleviate motor symptoms of PD [121]. Currently, l-DOPA still maintains the forefront of dopamine replacement therapy over 40 years of its development [122]. Although l-DOPA has been in use for over four decades as a primary line of pharmacological intervention, there are some highly noticeable adverse effects. One of the primary side effects is dyskinesia [123]. Other well-known side effects of l-DOPA include psychosis, hallucinations and other psychiatric manifestations [124]; and hypotension [125]. While replacement of the dopamine deficit with levodopa is an accepted treatment for PD, only ~25% of patients treated with this agent for 5 years continue to enjoy a good response; the remainder suffers from motor fluctuations and involuntary movements [126,127]. Attempts to discover drugs that not only ameliorate the symptoms of PD but also slow the time course of the disease have not been very successful. Sinemet (Carbidopa-Levodopa) and rasagiline have been promoted recently as two of the most suitable therapies to ameliorate the motor symptoms of PD. Sinemet, which is a combination of Carbidopa, and Levodopa, a precursor of dopamine, has become more popular in terms of managing motor symptoms with reduced occurrence of dyskinesia [128]. Rasagiline, a selective monoamine oxidase B inhibitor, has also been promoted because of its potential for reducing symptoms but recently a wide variety of reported side effects are causing concern about the use of this medication [129]. Additionally, various other dopamine receptor agonists such as pramipexole and rotigotine have become more frequently used as a combination therapy because they do not depend on enzymatic activation and have long lasting therapeutic effects [128]. These receptor agonists provide a line of defense against l-DOPA mediated changes in the movement of the arms and limbs and dyskinesia and help reduce the prescribed dose of l-DOPA. Although such agonist therapies are beneficial, various adverse effects such as hypotension, nausea, hypersexuality, hallucinations, and confusion can occur [130]. Another group of dopamine related combination pharmacotherapeutic agents to compliment l-DOPA are a family of catechol-*O*-methyltransferase (COMT) inhibitors. Commonly used COMT inhibitors, entacapone and tolcapone, work by inhibiting peripheral degradation of l-DOPA; thus increasing its half-life, stability and bioavailability [131].

Although there are a variety of dopamine replacement therapies available, alternative non-dopaminergic treatments are also gaining momentum to manage various symptoms of PD. One such approach is targeted towards basal ganglia where a restoration of balance between cholinergic and dopaminergic inputs can be achieved using anticholinergic drugs such as tri-hexyphenidyl and benztropine. These anticholinergics formed the basis of PD therapy before the introduction of l-DOPA [132] and were used to manage tremors. However, severe adverse effects, especially in elderly patients, limited their use. The adverse effects included cognitive impairment, constipation and urinary retention issues [133]. Various other cholinesterase inhibitors, such as donepezil and rivastigmine, are also currently being studied as therapies for PD. These drugs act by prolonging the effects of acetylcholine in cholinergic pathways by slowing down its metabolism [134,135].

### 5.2. Neuroprotection by Using Dopamine Receptor Agonists

These dopamine receptor agonists were proposed for use earlier than neuroprotectant and they act on D2 receptors which are commonly inhabited in terminals of dopamine synthesizing neurons of midbrain substantia nigra and eventually prevent any free radical induced cell damage. It has been shown that the dopamine receptor agonist can protect against degeneration of TH neurons in both *in vitro* and in animal models [136,137,138,139,140]. One such D2 receptor agonist is pramipexole, which has the ability to act as an antioxidant due to its chemical structure [137,140].

Recently, a couple of relatively big studies have investigated the use of dopamine receptor agonists to evaluate their ability protect neurons by using neuroimaging techniques in human PD subjects [141]. Although it seems that dopamine receptor agonists are neuroprotective, the main shortcoming of the study is that it is not possible to know whether the technique that has been used to evaluate with imaging can correlate neuroprotection of dopaminergic systems and not due to subtle chemical modifications of the radioactive tracers [142,143].

### 5.3. Therapeutic Potential of Antioxidants

Once it is recognized from the *in vivo* and *in vitro* studies that free oxy radical induced stress plays a central role in inducing Parkinsonism, a plethora of therapeutics have been proposed, that included selegiline, vitamin E, and rasagiline. Selegiline acts by reducing oxidation of dopamine by inhibiting synthesis of monoamine oxidase B (MAO-B). The MAO-B has been used in the human PD subjects. Recently, a DATATOP study was conducted to evaluate the putative therapeutic ability of selegiline along with vitamin E in subjects that showed early symptoms of PD [144,145]. The study revealed that selegeiline may delay the disease progression.

Rasagiline is another MAO-B inhibitor that also prevents free oxidative radical induced damage. This MAO inhibitor was also investigated in human PD from a different perspective of the disease [146]. This study suggests that PD patients when treated with raragiline early can have better effect in reducing the motor deficit.

Another agent that confers neuroprotection at least in animal models is coenzyme Q10 [147]. A small study involving PD patients reveals that Q10 administration resulted change in UDPRS score. This study also indicates that decrease in UDPRS in PD subjects that received CoQ10 was comparable to the control subjects that received placebo (*p* = 0.09) and beneficial effects was observed in small cohorts of subjects that received 1200 mg of CoQ10 [148].

Creatine has been shown to have neuroprotective effects and promotes mitochondrial ATP production in animal models [149]. A small study was conducted involving more than 50 patients that were diagnosed with Parkinsonian syndrome and given either creatinine or vehicle for over two years and the study suggests that there is no statistically significant change in UDPRS level between the two groups. Thus, as envisioned previously, none of the above mentioned neuroprotective agents showed huge promise towards mitigating PD pathology or slowing disease progression, but all of these antioxidants are safe. A more comprehensive study involving a larger number of patients with higher doses may give more insight about the utility of antioxidant therapies.

### 5.4. Apoptotic Inhibitory Factors

Although there are several agents that are known to inhibit apoptosis in *in vivo* models, TCH346 is the only such agent that recently been evaluated in human trials. TCH346 has the ability to prevent apoptosis by inhibiting a glycolytic enzyme. This agent has showed its neuroprotective potential in *in vivo* models of PD. However, a double blinded randomized human PD clinical trial using this agent did not show any positive result [150]. Likewise, CEP-1347 showed promise in animal models [151,152,153]; however, when evaluated in PD patients in clinical trials, it did not show significant improvement in either resting tremor or cognitive deficit [113]. The failure of these two clinical trials raised several important questions. Both the anti-apoptotic agents showed great efficacy in the preclinical neurotoxin induced PD models raising questions about the validity of the animal models. This also raised the question as to whether anti-apoptotic therapy is more useful when clinically relevant symptoms are evident and/or whether this kind of the therapy can be supplemented with other factors to get the full benefit of such therapy (157).

### 5.5. Trophic Factors

Although some factors which are commonly referred to as neurotrophic have shown promise in conferring neuroprotection in animal models, very few have been investigated in human PD patients. One such trophic factor is glial cell derived neurotrophic factor or GDNF that nourishes dopamine synthesizing neurons of the substantia nigra [116,154]. In human trials, GDNF was infused directly into the brain but a large clinical trial was not continued due to lack of any positive outcome [120]. This study raised concerns that the patients developed an antibody against GDNF. Subsequently, a study was conducted in monkeys where large dose of GDNF was administered and significant cell loss was observed in the cerebellum, suggesting an unsafe feature of this trophic factor.

Recently, gene therapy approaches involving neurturin, another neurotrophic factor, showed the ability to increase the longevity of the dopamine containing neurons in *in vitro* [155,156]. When the viral vector mediated method was used that delivered neurturin in the MPTP-treated monkey [157] or in 6-OHDA-treated rats [117], it protected against dopaminergic cell loss. This agent has been investigated in a human trial with a small sample size; the initial results showed substantial clinical effects and a larger double blinded clinical trial is underway [158].

Neuroimmunophilins are proteins that have strong affinity to immunosuppressive drugs and also present in great amount in the brain and induce growth of the neurons [159,160,161]. A study suggests that neuroimmunophilin ligands can protect dopaminergic neurons in the brain in PD models although the mechanism of action was not fully elucidated but the role of neurotrophic factors or glutathione could not be ruled out [162]. Recently a small clinical trial was conducted using the neuroimmunophilin ligand, GPI-1485, and the outcome was negative [163].

## 6. Drawbacks of Neuroprotective Therapy

Although several strategies showed promise in preclinical studies, none of the studies involving human PD patients showed any significant effects on the disease. Although the investigations that were conducted on humans were based on diverse mechanisms that lead to PD pathogenesis, some may have the potential to protect dopaminergic neurons of substantia nigra. However, collective failures of the clinical trials using putative neuroprotectant raise several questions about the testing methods as well.

### 6.1. Limitations of Testing Methods

The important issues are how to know whether an agent is neuroprotective in subjects inflicted with PD and what are the methods one can use to detect the efficacy of those agents in human. The common practice to date is to perform a neurological exam to evaluate the improvement of PD patients. Although the UDPRS system has been quite commonly used to monitor motor function, this system is not foolproof and there are several flaws in the UDPRS scaling system [164,165]. Patients who are also taking some other treatments along with the neuroprotective agents may have some effect on the UDPRS scale system that may mask the effect of neuroprotective agents. Moreover, the UDPRS scale is mostly rely on motor deficits and none of the autonomic dysfunction has been considered. A newer version of UDPRS scale is currently being developed that will not have the shortcomings of the existing scaling system [166].

“Confounding symptomatic effects” of the treatment are often encountered in human trials and to overcome this situation, many trials these days incorporate a time frame during that period administered drug will be washed away from the system before evaluating a patient, although the drugs which PD patients have been taking during neuroprotective therapy can have long lasting effects and may interfere with the final outcome as well [167]. Another method has been considered where one group will receive drugs earlier than the other [167,168]. Thus, the effect of the symptomatic treatment would be similar in both the groups. However, this approach is also not flawless [146] because a prolonged dosing method can initiate drug sensitivity. It is debatable whether this method is best to evaluate neuroprotection as PIs are afraid that patients may drop out during the trials.

### 6.2. Shortcomings of Animal Models

One of the major issues in devising neuroprotective therapies is limitations of the *in vivo* models. Currently, there are no available animal models that will mimic the full spectrum of PD pathology. Commonly, *in vivo* studies have been conducted in chemically induced models such as 6-OHDA or MPTP. These *in vivo* models display significant TH loss in the substantia nigra of the midbrain; however, the amount of time it takes to destroy dopaminergic neurons and pathogenesis is not similar to PD patients [169,170]. Recently, genetic models have been used in various studies to evaluate the role of different neuroprotective agents that have the ability to prevent DA degeneration. Although those genetic models add value to the studies with additional features, they still fall short of a model that will encompass all the features of this debilitating neurodegenerative disorder. Due to variation in displaying several important features of PD such as a-synuclein inclusion or TH loss and inability to show significant loss of TH neurons in the substantia nigra, these genetic models are not suitable for PD study [168,171].

At this time, most of the neuroprotective studies are being carried out on variety of *in vivo* models and we hope that results from these studies will pave the way for future clinical trials in humans involving PD patients.

### 6.3. Diversity of the Disease

PD is very diverse as genetic studies revealed that PD is not only associated with destruction of dopaminergic neurons of SN but there are some other causes that could lead to PD as well. Studies suggest that most of the point mutations are extremely rare and not likely to contribute any major role in PD pathogenesis although parkin mutations are quite prevalent in early onset of PD [71,172,173] and LRRK2 mutations contribute to cause 2% of the sporadic aspects of PD [174,175]. We cannot rule the possibility that a particular gene pool who are vulnerable to PD may contribute to the outcome of clinical trials involving neuroprotective agents against PD. Thus, it is important to know the genetics of the PD population to increase the chance of having a positive outcome of a clinical trial. Moreover, it is currently established that PD involves more than one mechanism that leads to behavioral manifestation of the disease, and it is possible that we may have to use combination therapy to inhibit those diverse mechanisms that lead to PD in humans.

## 7. The Future of Neuroprotection

### 7.1. Adenosine Receptor Antagonists

Another potentially attractive target as a non-dopaminergic therapy is the adenosine A_2A_ receptors because of their abundance in the striatal region. A_2A_ antagonists such as istradefylline and preladenant are in phase III studies as they have been regarded as an efficient line of therapeutics for reducing motor fluctuation. Although non-dopaminergic pharmacotherapy remains an essential aspect of the disease to mitigate PD pathology, to date no neuroprotectant has been found to be better than l-DOPA. However, additional therapies to l-DOPA, a reduction in motor function with adenosine A_2A_ antagonism, balance and gait improvement with acetylcholine and noradrenaline and reduction of dyskinesia were observed when aimed at glutamatergic or serotonergic systems as these studies have indicated a major platform for non-dopaminergic therapies of PD [176].

### 7.2. Anti-Inflammatory Agents

Although the role of dopamine and its down regulation has been recognized since the inception of the term “Parkinson”, not much attention has been paid to the role of inflammation; it is only recently that scientists have started to recognize the importance of inflammation in this neurodegenerative disease. Several aspects of the disease such as macrophage activation, pro-inflammatory cytokine production, and dramatic increase of CD molecules have been well documented in human inflicted with PD [80,81,82]. To mitigate the disease pathology, anti-inflammatory agents including that includes non-steroidal anti-inflammatory drugs and minocycline have been investigated to ameliorate PD associated symptoms. Studies conducted in both *in vitro* and *in vivo* models demonstrated that certain NSAIDs, *i.e.*, aspirin can protect dopaminergic neurons and ameliorate disease associated symptoms, however, controversy exist about the dose level and timing that provide the best neuroprotection [80]. In human clinical trials, it has been shown that the risk of having PD can be decreased by around 45% if NSAID has been taken regularly [96] and a subsequent study carried out by the same investigators showed that ibuprofen could be used as a neuroprotectant [177]. Currently, it is unclear whether available NSAIDs could really have any ameliorative effect in PD but focus should be given on developing drugs/agents that will target to prevent neuroinflammation could eventually halt or slow down the progression of PD in humans.

It has been few decades since statins have been introduced in the medical field that are known to reduce cholesterol; however, they are also able to prevent inflammation associated with PD by inhibiting pro-inflammatory cytokines and free oxy radical production [178,179]. In animal models, it has shown recently that Simvastatin can prevent degeneration of dopaminergic neurons in a chemically induced model of PD [178]. Subsequent epidemiological study has demonstrated that people who are taking simvastin can lower the risk of having PD [98,180]. Recently it has also been demonstrated that low levels of LDL cholesterol can elevate the risk of having PD [181,182], thus it is possible that if we increase the statin level it may mean that greater the level of LDL, lower the risk of PD, again indicating the neuroprotective aspects of the drug.

Recently it was shown that minocycline which is commonly used to prevent growth of harmful microbes, possess some of the property to protect cells from inflammation induced injuries in the brain. Recent study revealed that Minocycline inhibits the activation of macrophage in the brain and also can prevent apoptosis in cell culture [183]. Minocycline has found to be neuroprotective in chemically induced *in vivo* models of PD [184,185,186]. Subsequently, minocycline has shown promise in human study as humans can tolerate this drug without much side effects leading to larger study in phase III clinical trials [99].

### 7.3. Other Antioxidants

Recently conducted clinical trials on humans using uric acid suggests that it can be used to ameliorate PD. Basically, uric acid can prevent generation of oxidative free radicals and free nitrogen species to prevent further oxidative stress [187]. Recently, it has been demonstrated that people who have high levels of urate in their serum have low risk of having PD. In patients with early PD, a high level of uric acid is associated with slower disease progression [188]. *In vitro* studies suggest that uric can prevent degeneration of dopamine neurons when exposed to toxin, rotenone [183]. Recently, it has been demonstrated that subjects who are on a diet that will generate greater amount of urate could decrease the risk of having PD, suggesting that uric acid can serves as neuroprotective therapy in PD [189]. A large scale study involving human PD will be needed to show effectiveness of elevating urate levels in the blood.

### 7.4. Strategies to Use α-Synuclein as Neuroprotectant

It is not well documented how α-synuclein induces neurotoxicity, but it seems to be an important molecule in inducing pathogenesis of PD. Thus, prevention of the aggregation of this misfolded protein should be the main target in developing neuroprotective therapies. There are several ways one can prevent accumulation of these misfolded proteins; first, by inhibiting the accumulation and or synthesis of α-synuclein, second, by expediting the process of clearance of α-synuclein, and third by inducing changes in the intermediate chemical molecules that are responsible for synthesis of α-synuclein. A recent molecular biological approach infers that we can increase the clearance of α-synuclein by activating ubiquitin proteasome pathways. An increase in the level of parkin or UCH-L1 can help increase the clearance of α-synuclein as recent evidence suggests that overexpression of parkin can prevent aggregation of α -synuclein in *in vivo* models of PD [190,191,192]. Chaperones such as hsp 70 can also promote α-synuclein clearance and also have the ability to reduce insoluble α-synuclein accumulation both in *in vivo* and *in vitro* models of PD [75] and geldanamycin can reduce the α-synuclein aggregation *in vitro*. Recently, it has been demonstrated that activation of lysosomal pathways can also reduce α-synuclein accumulation in the cell and the lysosomal enzyme cathepsin D can decrease α-synuclein accumulation both in *in vivo* and *in vitro* models of PD [193]. Studies also suggest that α-synuclein transgenic mice when vaccinated with α-synuclein displayed dramatic decrease of aggregated α-synuclein. [194]. Recent evidence also suggests that chemical changes such as oxidation and phosphorylation are essential for accumulation of misfolded protein, α-synuclein [195,196,197]. Thus, it is tempting to speculate that strategies targeted at oxidation and inhibitors of kinase activity could be useful in preventing α-synuclein aggregation.

### 7.5. Kinase Inhibitors

We have shown that a prominent non-receptor tyrosine kinase, c-Abl, regulates several cellular processes that may be linked to PD [198]. Several studies followed our findings and have shown the involvement of c-Abl in neurodegenerative diseases such as PD using various *in vivo* models of PD. Moreover, the c-Abl protein level is found to be elevated in the postmortem striatum of PD patients [199,200]. These studies also reinforce a report of increased c-Abl phosphorylation at Y412 in the substantia nigra [199,200] and striatum [200] of PD patients. In the earlier study, c-Abl was found to phosphorylate parkin and impair its E3 ligase activity, and eventually degenerate dopaminergic neurons of the SN [200], a finding reproduced by Imam *et al.* (2011). More recently, Hebron *et al.* [199] have demonstrated that c-Abl has the ability modulate the clearance of α-synuclein. These extraordinary findings led to the development of an efficient therapeutic target for PD, thus opening the avenue for the repositioning of c-Abl inhibitors that are already in the clinic or being used as orphan drugs for certain types of leukemia. Thus, when we inhibit the c-Abl activity by imatinib/Gleevec [201], nilotinib/Tasigna [202] or bafetinib/INNO-406 [203], they can bring DA to normal amounts and protect the degeneration of TH loss in the midbrain SN of mice [199,200].

### 7.6. Trophic Factors

One of the older approaches to treat patients who are inflicted with Parkinsonian syndrome is trophic factors. The main advantage of this strategy is that nature of this factor and their mode of action is well known. Another aspect of these trophic factors makes them ideal candidates for neuroprotection is that they do not rely on mechanisms of cell death. Conversely, these trophic factors have the ability to act directly on the loss of TH in the brain without affecting non-dopaminergic part of PD [50].

## 8. Possible Therapeutics for Nonmotor Symptoms of PD

Although the main motor symptoms in PD are apparent once the majority of the DA neurons in the SNpc are lost, we cannot ignore the nonmotor symptoms associated with this debilitating neurological disorder. Nonmotor symptoms are basically neuropsychiatric, autonomic, sleep and sensory. These symptoms are prevalent in most of the PD patients and predominantly seen in PD patients who survive with the disease for a long period [204]. Although Clozapine has shown promise to mitigate the nonmotor symptoms seen in PD, due to its possible cause of agranulocytosis, another drug called quetiapine has been introduced as a better antipsychotic drug. Variable degrees of depression have been observed among 40%–70% of the PD patients with a smaller number of PD patients suffering from major depressive disorder [205]. Both cognitive deficit and dementia are prevalent in PD. While dementia occurs mostly at the later stages of PD and may cause a significant impact in social and occupational functioning, cognitive deficits can be seen early in PD but do not affect social or occupational activities. Although it is reported that dementia can affect 30%–40% of patients inflicted with PD, recent studies suggest that the frequency of depression can be as high as 80% [205]. Although recently a comparative study was conducted for tricyclic antidepressants (TCA) *vs.* selective serotonin reuptake inhibitors (SSRIs) from the efficacy standpoint, the data was quite difficult to interpret due to the low power of the studies. Only one human study involving 52 PD patients with depression suggests that TCA nortriptyline, which is a non-specific norepinephrine reuptake blocker (SNRI), seems to be more efficacious than the SSRI paroxetine CR in reducing depression in PD [206]. Thus, it is essential to develop biomarkers and putative neuroprotective agents aimed at nonmotor symptoms and eventually manage the course of this debilitating disease.

### 8.1. Surgical Therapies for PD

Although stereotactic surgeries targeting internal globus pallidus and ventro lateral thalamus were commonly used in the 1950s and 1960s to treat PD patients, these procedures lost favor with neurosurgeons after the introduction of levodopa as better pharmacotherapy for PD. Recent ablation studies in animal model of PD and the introduction of the deep brain stimulation technique revolutionized our ability to comprehend about the pathogenesis of PD [15,16,17,207,208]. In the ablative surgeries, two loci such STN and GPi were targeted. However, due to cognitive and psychiatric adverse effects, the ablative procedures are not used in developed countries since DBS procedures have become more popular. In the DBS procedure, electrodes are implanted into either STN or GPi that has advance system connected to imaging and electrophysiological techniques. This unique procedure receives electrical stimulation in the implanted brain areas at a continuous rate. These procedures are best suited for PD patients that have previously improved on levodopa and are free of dementia, or other psychiatric co-morbidities [209,210]. It has recently been documented that DBS performed either unilaterally or bilaterally improves parkinsonian motor signs [209,211,212,213,214].

### 8.2. Are There Any Non-Motor Side Effects of STN and GPi-DBS?

Although DBS involving either targeting GPi or STN are found to be effective, they are not free of adverse effects. Both these procedures may some side effects on communication ability and cognitive deficit [215,216,217,218,219,220,221,222,223]; although verbal fluency and cognitive deficits were more commonly seen in older patients [222,224]. Since DBS is commonly used to treat PD patients even in the early stages, one should account for all the possible adverse effects. Recent studies suggest that DBS may cause manic depression, induce emotional instability and suicidal thoughts [221,223,225,226,227,228,229,230].

### 8.3. Is STN a Better Target than GPi for DBS to Treat PD?

Although there is a perception that STN could be a better target than GPi, recent large randomized clinical trials comparing GPi and STN-DBS did not find any significant difference in terms of motor side effects of the procedures [211,231]. Another recent study suggests that patients who have undergone STN-DBS have a better health-related quality of life; however, occupational function or interpersonal relationships do not improve [232]. Thus, it is possible that patients as well as neurosurgeons may prefer GPi-DBS over STN due to the lower chance of side effects.

### 8.4. Possible Neural Transplantation for PD

In 1980, cell transplantation into the brain was considered a potential cure for PD. Although a plethora of studies in animal models of PD and clinical trials where fetal dopaminergic neurons were injected in the caudate putamen showed promise [20], recent clinical studies have several concerns about the safety of this method for treating PD [21,22]. In the study involving human PD subjects, no significant improvement was noticed and graft-induced medication-independent dyskinesia developed [22]. Recently, it has been shown that PD subjects who died 10–15 years after the transplant have α-synuclein in the transplanted cells, indicating that implanted cells were also affected by the neurodegenerative processes in the patient’s brain [23,118,233]. Alternatively, another group of studies suggest that graft-induced dyskinesia was due to implantation of serotonergic neurons into the grafts that may have dysfunctional cells that either did not release dopamine regularly or acts as a pseudo-transmitter for serotonergic terminals [234,235]. This notion was further confirmed by another set of experiments where it has been shown that chronic injection of buspirone, decreases neuronal activity of serotonin, and ameliorates transplant related dyskinesia in patients inflicted with PD and also in rat models of PD [234,235,236] although the efficacy in preventing PD was undetermined.

### 8.5. Gene Therapy for PD

Recently, a group of scientists have proposed a method where a viral vector has been used which could be useful in treating PD patients [237,238,239]. Basically, this method makes neurons produce neurotransmitters which are chemically different. There are two anatomical areas that have been targeted to evaluate: one is STN where the AAV vector could be used to infuse enzyme GAD which has the ability to transform neurons that are glutamatergic in nature into GABA synthesizing neurons [240,241], and another region is the caudate putamen where this viral vectors can be infused to differentiate into striatal neurons that eventually synthesize neurotransmitter dopamine [242,243,244]. Evaluation of the efficacy of both the methods described above is under investigation in human clinical trials. The use of gene therapy is still debatable due to safety concerns. It is not clear how to ensure proper use of this method as it is not known whether this method producing neurotransmitters using viral vector will produce enough amount or whether one can reverse the process to adjust the required amount that PD inflicted person need.

## 9. Future Directions: Search for Biomarkers and Neuroprotective Therapies

So far we have discussed the progress made over the past several decades in the development of drugs to treat symptomatic PD. However, one should consider the number of important challenges that neurologists and PD researchers face in the quest to develop an early detection method based upon novel biomarkers that will enable them to recommend the most effective neuroprotective treatments. Currently, most of the PD symptoms appear when 70%–80% of the dopaminergic terminals are lost in the striatum and more than half of the dopamine synthesizing neurons lost in the substantia nigra of the midbrain, thus early detection and intervention is crucial for effective neuroprotective treatment which may prevent the degeneration of the Dopaminergic neurons and eventually PD pathogenesis. Recent failures of several neuroprotective clinical trials [22,34,245,246] again ushers the importance of biomarkers and their use in early detection of the disease.

### 9.1. Can Olfactory Dysfunction Used as an Early Biomarker?

It has been 30 years since the association between olfactory dysfunction and PD was first reported [247], and since then a plethora of studies have been conducted and some studies suggest that anosmia pay precede the onset of motor disease [248,249,250]. Varying degrees of olfactory dysfunction have been reported in 50%–90% of PD patients but there is little correlation found between anosmia and the clinical severity of PD [251,252,253,254]. There are conflicting reports available regarding the substrate of olfactory loss. While a few studies suggest degeneration of olfactory epithelium of morphological change of olfactory bulb is the main reason of dopaminergic degeneration [255,256,257], others demonstrated that dopamine synthesizing neurons increased in the olfactory bulb, especially in the periglomerular region of patients inflicted with PD [248,249,250,258]. Numerous studies in the last decade have alluded to the fact that PD patients have early anosmia, thus suggesting that it could be used as an early biomarker to detect PD [259]. Additionally, this method could also be exploited to demarcate from other neurological diseases such as progressive supranuclear palsy (PSP) or corticobasal degeneration (CBD) but not from multiple system atrophy (MSA) [255,260,261]. Recently, the American Academy of Neurology recommended using olfactory function screening method to differentiate PD from PSP but not from MSA [262]. It is important to note that anosmia is not only seen in PD but also prevalent in Lewy body dementia, in AD patients, and in aging population, and could also be side effects of medicines patients consume [263,264,265,266]. Therefore, olfactory testing used in conjunction with other biomarkers and imaging techniques may serve as a better tool for early detection of PD and thus earlier intervention with neuroprotective therapies before the emergence of extensive dopaminergic neuronal loss and motor symptoms [249,250,267].

### 9.2. Can Neuroimaging Possibly Be Used an Early Biomarker?

Presently, three main imaging methods, positron emission tomography (PET), single photon emission computed tomography (SPECT) and magnetic resonance imaging (MRI) are routinely used to detect PD pathology. PET and SPECT are two methods used to detect pathology in PD patients as these procedures identify and map the changes in the abundance that can be extrapolated as a dopamine synthesis in the caudate putamen [245,268]. Among PET and SPECT imaging procedures, PET is less favored due to its excessive cost; however, SPECT dopamine transporter (DAT) imaging is more popular as it is cheaper and sensitive to screen dopamine loss in the striatum [269]. In this regard, there are various DAT ligands that have been developed and used to evaluate PD patients such as 123Ioflupane. The main advantage of DAT imaging is that it could be used as an early detection tool to identify early dopaminergic loss in PD patients. One must keep in mind that the SPECT DAT imaging technique should be used in combination with some other symptoms such as anosmia, cognitive deficits, cardiac disorder and insomnia to evaluate the patients that have not been diagnosed [270,271,272]. This combined approach (SPECT DAT imaging with smell loss) has been used recently to identify individuals who have a high risk of developing PD [273,274]. Longitudinal clinical and imaging evaluations will be beneficial in identifying and assessing the progression of deficits; state of the art DAT imaging in prospective PD patients and subsequently neuroprotective therapies could be used to slow or halt the neuronal degeneration and eventual onset of symptomatic PD [267]. In this regard, neuroinflammation, oxidative stress, α-synuclein deposition, and protein misfolding could be combined with neuroimaging. Recent studies showed that imaging techniques that target activated microglia are certainly upregulated in the human brain that are inflicted with PD [275,276,277] although the efficacy and usefulness of this approach will rely on specificity and sensitivity of the ligand targeted for different markers.

### 9.3. Can Proteomics and “Omics” Approach Used as Biomarker?

An array of different, sensitive omics’ methods has been developed to identify relevant pathways for PD therapeutics [278,279]. The use of these various omics approaches at different levels such as “transcriptomics” that reflects change in gene expression, “proteomics” that evaluates changes in the abundance of proteins or “metabolomics” which refers to changes in metabolites in biological specimens such as brain tissue, CSF or in blood may help us identify pathways that are dysfunctional in PD. All these omics approaches may help us to delineate pathways such as oxidative stress or axon guidance which could be playing a role in the degeneration of degeneration of neurons in the brain. The information gathered from these “omics” approach may provide a better understanding of the underlying cause and plausible signaling mechanisms involved in human PD. Metabolomics can be defined as measurement of different metabolic responses of *in vivo* systems which eventually trigger or modify genetic content of an organism [280] and the response can be influenced by internal influences such as the gut microflora and external influences such as diet, exercise, *etc.* [281]. This advanced “omics” approach largely depends on spectroscopic platforms such as NMR and mass spectroscopy (MS) coupled with liquid chromatography (LC) to evaluate biological samples (e.g., urine and serum). The metabolomics approach can provide information regarding the pathophysiological status of an *in vivo* system [282]. This approach could be exploited to evaluate various diseases including Huntington’s [283]. Using gas chromatography (GC)/MS-based approach, Michell *et al.* investigated different metabolites in serum and urine samples from human PD inflicted subjects and their age matched controls [284]. This method indeed measured a subtle difference that may exist in human PD subjects compared to their age matched counterparts, and thus highlights the importance of this technique in early detection of PD in humans. In a separate follow up study, Bogdanov *et al.* have combined HPLC with electrochemical colorimetric method to evaluate possible biomarkers in plasma samples from patients inflicted with PD [285].

### 9.4. Stem Cell Approach to Treat Parkinson’s Disease

In 1987, the first clinical trial of cell transplantation was conducted in PD patients [286]. Initially, PD patients showed a dramatic improvement in their symptoms but modest changes in others. Later, larger studies revealed side effects in some patients that received similar grafts [287]. Neural stem cells (NSC) are mainly from the hippocampus and from the subventricular zone (SVZ) near the lateral. Although a huge effort was made using NSC to generate DA neurons in animal models [288,289], controversy persists regarding neurogenesis in the SVZ in the PD models. Thus, it is important that transplants of NSC justify their capability to improve the refined motor problems associated with PD and long term culturing of NSC and their stable differentiation potential is guaranteed. Embryonic stem cells (ESC) have the ability to develop any cell type of the body. The ESC may generate DA neurons at a specific developmental stage that is prior to or during the cell’s fate to a neural phenotype. By using stringent procedures, it is certainly attainable to differentiate dopaminergic neurons which have chemical phenotype that of human brain, moreover, this ESC can also withstand procedures involved in grafting and certainly have the ability to regain control of movement of limbs in *in vivo* models of PD [290,291,292]. Although there has been lots of promise based on preclinical studies, it is imperative to monitor the efficacy and their stability for longer duration. Incomplete differentiation of ESC is a major challenge for the development of an effective PD therapy and the risk of tumorigenesis may prevent the applications of ESC [287]. Induced pluripotent stem cells (iPSCs) are better sources for PD as these can be derived from the patients, thus eliminating the risk of immune rejection and the requirement of immunosuppressive therapy [293]. Unlike ESCs, iPSCs are not hindered by ethical issues [294]. Research demonstrated that generated iPSCs could be well differentiated into DA neurons of the midbrain, which subsequently have the ability to diminish motor symmetry in the 6-OHDA induced PD model [295]. Likewise, recent studies showed that transplantation of iPSC derived from human protein have the ability to restore movement of the limbs [296]. Thus, human iPSCc can provide a promising source of midbrain DA neurons for cell therapy for PD [297]. Mesenchymal stem cells (MSCs) have many salient features such as they can be easily extracted, cultured and expanded [298,299]. In animal studies of PD, it has recently been demonstrated that MSCs can prevent DA degeneration and restore motor deficits [300]. In a separate study, when BMSCs were infused into an *in vivo* rat model of PD, those BMSCs synthesized TH and restore motor deficits associated with PD [301]. MSC derived from the umbilical cord also shows neuroprotective ability in 6-OHDA models of PD [302,303].

## 10. Conclusions

In this review, we have tried to cover a broad range of strategies that could be used to prevent the progressive neurodegeneration seen in PD. Several theories have been proposed about whether the pathogenesis of PD and mitochondrial theory is still tenable based on the fact that a number of studies support this theory. The main point we would like to emphasize is that PD pathogenesis is not a result of dysfunction of one specific pathway but rather a combination of interconnected events contributing to pathogenicity [304]. Unequivocally, aging is by far the most common risk factor of PD and any modulators that can expedite the aging process are can also affect the risk for PD. Thus, it is important to find neuroprotective agents that can restore the DA levels in the caudate putamen and prevent or delay the degeneration of DA neurons in the midbrain and eventual motor symptoms associated with PD. Although levodopa revolutionized PD therapy in the early 1960s, non-motor symptoms associated with PD were not alleviated. Thus, it remains to be seen how researchers and neurologists will overcome the challenges to develop and characterize treatment modalities that will not only prevent dopaminergic loss but also help mitigate autonomic, psychiatric and cognitive dysfunctions in PD patients.

## Figures and Tables

**Table 1:** Mechanism of Parkinson’s disease (PD) pathogenesis and possible targets for therapy (adopted from Yacoubian TA *et al.*, 2009, [50]).

PD Pathogenic Mechanism	Targets for Neuroprotection
Oxidative stress and mitochondrial dysfunction	Inhibitors of dopamine metabolism (e.g., MAO inhibitors, dopamine receptor agonists)
	Electron transport enhancers (e.g., CoQ10)
	Other Oxidants (e.g., Vitamin E, Uric acid)
	Glutathione promoters (e.g., selenium)
	Inhibitors of a-synuclein aggregation
	Therapeutic agents that reduce α-synuclein protein levels
	Enhancers of parkin function
	Enhancers of UCH-L1 function
	Enhancers of proteosomal or lysosomal pathways
Protein aggregation and misfolding	Anti-inflammatory agents (e.g., NSAID, statins, minocycline)
	NMDA receptor antagonists, Calcium channel antagonists
	Anti-apoptotic agents
Neuroinflammation	Neurotrpohic factors (e.g., GDNF, neurturin)
Excitotoxicity	
Apoptosis and cell death pathways	
Loss of trophic factors	
